# The Predictive Effectiveness of Blood Biochemical Indexes for the Severity of COVID-19

**DOI:** 10.1155/2020/7320813

**Published:** 2020-08-07

**Authors:** Yingchu Zhou, Bo Li, Jiyang Liu, Dong Chen

**Affiliations:** The First Hospital of Changsha, The Hospital of Infectious Diseases of Changsha, The Public Health Treatment Center of Changsha, Changsha 410005, China

## Abstract

**Objective:**

We aimed to explore the predictive effectiveness of blood biochemical indexes for COVID-19 severity.

**Method:**

We retrospectively analyzed the clinical data of COVID-19 patients who were cured and discharged from the Public Health Treatment Center of Changsha from January 30, 2020, to February 19, 2020. According to the clinical classification of the disease, the patients were divided into severe and nonsevere groups. General clinical data and underlying medical conditions were recorded through the electronic medical record (EMR) system. Laboratory examination results of the patients during their hospitalization were collected, including the first results for erythrocyte sedimentation rate (ESR), C-reactive protein (CRP), peripheral blood lymphocyte ratio and count, and peripheral blood white blood cell (WBC) count. Univariate and multivariate logistic regression models were used to analyze the predictive effectiveness of blood biochemical indexes and other related factors for COVID-19 severity.

**Result:**

In all, 108 COVID-19 patients (median age: 43.9 years (range: 1–75); male patients: 56 (51.85%)) were enrolled, of whom 24 (22.22%) showed severe disease and 84 (77.78%) showed nonsevere disease, and two in 24 patients with severe disease developed into a critically severe type and died. Fever was the most common onset symptom (67.59%), followed by cough (48.15%) and fatigue (37.04%). Comorbidities were important factors affecting the severity of COVID-19, and among the patients with severe disease, the proportion with comorbidities was 70.83%, and the proportion without comorbidities was 29.17%. The intergroup difference was significant (*P* < 0.05). In patients with CRP levels (mg/L) of ≤8, >8–≤20, >20–≤40, and >40, the proportions of those with severe and nonsevere disease were 0 to 32, 7 to 19, 6 to 23, and 11 to 10, respectively; the intergroup difference was significant (*P* < 0.05).

**Conclusion:**

The presence or absence of comorbidities and CRP elevation were independent significant predictors of COVID-19 severity, and hypertension was found as the most common comorbidity in patients with severe disease.

## 1. Introduction

An acute respiratory infection caused by a novel coronavirus, severe acute respiratory syndrome coronavirus 2 (SARS-CoV-2), has been circulating in Wuhan, Hubei Province, China, since December 2019 [1]. The disease is currently endemic in many countries. Furthermore, there are reports of imported SARS-CoV-2 infection from abroad in many places in China [2]. Because of the rapid spread of novel coronavirus disease (COVID-19), a simple and effective indicator to assess the severity and determine the prognosis of new crown pneumonia is urgently needed at this stage. The classification of disease severity is very important for the classification of treatment of patients, especially in the case of an epidemic situation and relatively scarce medical resources. Furthermore, it is necessary to conduct treatment per classification, optimize the allocation of rescue resources, and prevent insufficient or excessive treatment. Retrospective analyses of clinical characteristics and blood biochemical indexes of discharged patients may help determine the clinical characteristics and severity of COVID-19. Studies in China and abroad have found [3–5] that patients infected with SARS-CoV-2 often have decreased peripheral blood lymphocyte counts, increased C-reactive protein (CRP) levels, and increased erythrocyte sedimentation rates (ESRs). Lymphocytes play a decisive role in maintaining the body's immune homeostasis and inflammatory response. Understanding the mechanisms underlying changes in these blood biochemical indexes is expected to provide an effective strategy for the treatment of COVID-19. Furthermore, the protection, maintenance, and promotion of lymphocyte counts may facilitate the prevention and treatment of new crown pneumonia. Since all the literature published on COVID-19 thus far is focused on hospitalized patients, with many patients still being under hospitalization or under treatment, we retrospectively analyzed the patients admitted to the Changsha Public Health Treatment Center, Hunan Province, China, who had been cured of COVID-19 and discharged from the hospital between January 30, 2020, and February 17, 2020. Because the clinical end point has been achieved in the case of these patients, we believe the prediction accuracy and reliability of this study are higher. Thus, we aimed to assess the predictive effectiveness of the patients' clinical characteristics and blood biochemical indexes for COVID-19 severity. We hope that the findings of this study can provide useful information for early decision-making and treatment for COVID-19.

## 2. Methods

### 2.1. Objects

The study was conducted according to the principles of the Declaration of Helsinki. The requirement of written informed consent was waived by our institutional review board because this was a retrospective study that evaluated deidentified data and involved no potential risk to patients and no link between the patients and researchers. In all, 108 patients (56 male and 52 female patients; median age: 43.9 years (range: 1–75)) diagnosed with COVID-19 who had been cured and discharged from the Public Health Treatment Center of Changsha between January 30, 2020, and February 19, 2020, were included. The patients' general clinical data and information about their underlying medical conditions were collected through the electronic medical record (EMR) system. Thirty-five patients (32.41%) had comorbidities, including hypertension (14.81%), diabetes (4.63%), cerebral infarction (3.7%), liver disease (including hepatitis b and fatty liver; 9.26%), and chronic respiratory diseases (2.78%).

### 2.2. Diagnostic Criteria

Diagnostic criteria were implemented according to the “the diagnosis and treatment program of new coronavirus pneumonia (trial sixth edition)” [6] which was updated in real time and released by the National Health Commission, China. The diagnostic criteria for severe diseases were also according to the abovementioned scheme and were as follows: (1) shortness of breath, respiratory rate (RR) ≥ 30 times/min; (2) at-rest oxygen saturation ≤93%; and (3) arterial partial oxygen pressure (PaO_2_)/oxygen absorption concentration (FiO_2_) ≤300 mmHg (1 mmHg = 0.133 kPa). A case is defined as severe if pulmonary imaging showed an obvious lesion progression of >50% within 24–48 h. Illness was judged a critically severe type if any of the following criteria were met: respiratory failure requiring mechanical ventilation; shock; and requirement of ICU care for patients with other organ failure.

### 2.3. Discharge Standards

Discharge standards were implemented according to the “the diagnosis and treatment program of new coronavirus pneumonia (trial sixth edition)”^6^.Patients were discharged if all of the criteria were satisfied: (1) normal body temperature for more than 3 days, (2) significant improvement in respiratory symptoms, (3) significant improvement in acute exudative lesions as indicated by pulmonary imaging, and (4) a negative nucleic acid test for two consecutive respiratory specimens (with a sampling interval of at least 24 h).

### 2.4. Observation Indicators

The patients' age, sex, comorbidities, epidemiology, and blood biochemical indexes were retrospectively analyzed. The patients were divided into groups according to their age. Epidemiology was defined in terms of the presence or absence of an exposure history associated with Wuhan. Cases were categorized as severe and nonsevere. The blood biochemical analyzed included the peripheral blood white blood cell count, peripheral blood lymphocyte ratio and count, ESR, and CRP. The relationships between major blood biochemical indexes and disease severity and between age, sex, epidemiology, comorbidities, and disease severity were analyzed to understand the effects of the abovementioned parameters on disease severity. The clinical early warning indicators in severe and critical patients were identified to provide a reference basis for clinical diagnosis and treatment and guideline formulation.

### 2.5. Statistical Analysis

SPSS 17.0 software was used for statistical description and analysis of test data. Continuous variables are expressed as medians or simple ranges (if appropriate). Categorical variables are summarized as counts and percentages. For all data, the Pearson chi-squared test and multivariate logistic regression were used to analyze the predictors of COVID-19 severity.

## 3. Results

Table 1 summarizes the clinical characteristics of the 108 patients included in the study.

Of the 108 patients, 24 (22.22%) had severe disease and 84 (77.78%) had nonsevere disease, and two in 24 patients with severe diseases developed into a critically severe type and died. The cause of death of one critically severe patient was secondary infection, septic shock, and multiple organ failure. Aspergillus growth was found in the blood culture of this patient on the third day after death. Another critically severe patient died from a storm of inflammatory factors triggered by SARS-CoV-2 which led to heart failure and repeated cardiac arrest.

Fifty-eight patients (53.7%) had a history of Wuhan-associated exposure (including long-term residence in Wuhan or travel to Wuhan within 14 days before hospitalization). Twelve patients (11.11%) had no clear related epidemiological history. The most common symptom was fever (67.59%), followed by cough (48.15%), fatigue (37.04%), sore throat (13.89%), muscle soreness (11.11%), expectoration (10.19%), chills (9.26%), and shortness of breath (7.14%). Rare symptoms included dizziness, headache, diarrhea, and vomiting. Among the patients with severe diseases, the proportion with comorbidities was 70.83% and the proportion without comorbidities was 29.17%. The most common comorbidities were hypertension (14.81%) and chronic liver disease (9.26%). In patients with a peripheral blood white blood cell count (×10^9^/L) of <4, ≥4–<10, and ≥10, the proportions of those with severe and nonsevere disease were 6 to 33, 17 to 48, and 1 to 3, respectively. The corresponding proportions in patients with a peripheral blood lymphocyte percentage (%) of <20, ≥20–≤40, and >40 were 11 to 16, 13 to 61, and 0 to 7. In patients with peripheral blood lymphocyte counts (×10^9^/L) of <0.8, ≥0.8–≤4.0, and >4.0, the proportions of those with severe and nonsevere disease were 11 to 14, 13 to 68, and 0 to 2, respectively. Among patients with a C-reactive protein level (mg/L) ≤8, >8–≤20, >20–≤40, and >40, the proportions of severe and nonsevere cases were 0 to 32, 7 to 19, 6 to 23, and 11 to 10, respectively. Among patients with ESR (mm/h) ≤40, >40, ≤80, and >80, the proportions of severe and nonsevere cases were 5 to 49, 13 to 26, and 3 to 7, respectively.

The data obtained by the chi-squared test showed that patient age >40 years, combined with basic diseases, decreased peripheral blood lymphocyte percentage and count, increased C-reactive protein level, and increased blood sedimentation were all factors related to COVID-19 severity (all *P* < 0.05). With the increase in age, especially in patients older than 65 years, the proportion of patients with severe diseases increased significantly. The incidence of severe disease in patients with comorbidities was significantly higher than that in those without comorbidities (*P* < 0.05). Increased serum sedimentation and C-reactive protein levels were positively correlated with the severity of the disease. Among patients whose peripheral blood lymphocyte percentage or (and) count was below normal, the incidence of severe illness is greater, However, patients with increased lymphocyte ratio and counts did not show severe illness. Factors with *P* < 0.05 in the univariate analysis were included in the multivariate logistic regression model, but only the underlying disease (*P*=0.004) and increased CRP level (*P*=0.005) were associated with the disease. The findings suggest that CRP elevation and a previous history of comorbidities are important clinical characteristics and independent predictors of severe COVID-19. Moreover, the elevation of CRP levels was positively correlated with the severity of the disease. In one case, the patient died while in the hospital, and as the illness got worse, both the peripheral blood lymphocyte ratio and the count showed a progressive decline. The lymphocyte percentage and count reached the lowest points of 0.9% and 0.08 × 10^9^/L, respectively, when the disease rapidly deteriorated; these values were significantly lower than normal. The chi-squared test and multivariate regression analysis did not show correlations between disease severity and gender, Wuhan exposure history, contact with confirmed patients, and peripheral blood leukocyte count level. There was no statistical significance in the SPSS 17.0 software analysis in severe and nonsevere patients (*P* > 0.05).

## 4. Discussion

In December 2019 [7], an outbreak of pneumonia was reported in Wuhan, Hubei province, China. As the epidemic spread, cases have also been reported in other parts of the country and many other countries. Through a series of medical prevention and treatment measures, the upward trend of the epidemic in China has been controlled to some extent, but there have been outbreaks in foreign countries [8]. Prompt summarization of the clinical characteristics of patients who reached the end of clinical treatment will be of great value in predicting the severity and prognosis. The pathogen has been identified as a novel enveloped RNA betacoronavirus, and the pathological features of COVID-19 are very similar to those of Middle East respiratory syndrome (MERS) and SARS [9]. The world health organization (WHO) officially named the disease “coronavirus disease 2019 (COVID-19)” on February 11, 2020. The main source of infection seen so far has been patients infected with the new coronavirus, although asymptomatic patients can also be a source of infection, which mainly spreads by respiratory droplets and close contact through aerosol transmission in a closed environment for extended periods. The virus was also isolated in urine, feces, and other body fluids. Based on the current epidemiological data, the incubation period of the virus is 1–14 days, with an average incubation period of 4 days (mostly 2–7 days) [5]. Fever, dry cough, and fatigue are the main manifestations of this disease, while a few patients also show nasal congestion, runny nose, sore throat, myalgia, and diarrhea. Our study confirmed these characteristics, but some patients showed no initial signs of fever or radiological abnormalities, making early diagnosis of COVID-19 extremely difficult [5, 10]. Most of the patients with severe disease had dyspnea and/or hypoxemia one week after onset [5, 11, 12].

For COVID-19, an important, but difficult, objective of treatment is to avoid the development of severe or critical disease in the nonsevere patients. In this study, age ≥40 years, presence of complicated underlying diseases, peripheral blood lymphocyte count <0.8, peripheral blood lymphocyte ratio <20%, ESR >40, and CRP >20 were all early warning signs of severe illness. In one case, with the aggravation of the disease, the peripheral blood lymphocyte ratio and count showed a progressive decline during the hospitalization period. The lymphocyte percentage and count reached the lowest points of 0.9% and 0.08 × 10^9^/L, respectively, when the disease rapidly deteriorated, which were significantly lower than normal. Thus, the findings of our study indicate that, for patients showing an early reduction in the peripheral blood lymphocyte percentage or count, CRP levels greater than 40, or an ESR elevation greater than 20, strict monitoring should be performed to achieve early prevention, detection, and intervention. Moreover, for these patients, aggressive treatment should be initiated to avoid deterioration of the disease and progression to a severe or critical status and to reduce the case fatality rate in severe patients. The Shanghai protocol [13] suggested that thymosin could be injected subcutaneously twice a week since it showed the ability to improve the patient's immune function, thereby preventing the disease from becoming severe and shortening the period of detoxification. On a related note, the seventh edition of the guidelines of the national health and fitness commission [7] advocates the integration of traditional Chinese and Western medicine and the individualized treatment of patients with traditional Chinese medicine.

Nevertheless, in a multivariate logistic regression analysis, the effect of early lymphocyte levels on the severity of disease was not statistically significant, which may be related to the small sample size of this study. Multicenter clinical studies with larger sample sizes are required to confirm this conclusion. For patients with a decreased peripheral blood lymphocyte count and ratio, increased ESR, or increased CRP levels, especially those with an ESR increase of more than 40 and a CRP increase of more than 20 or those meeting all three criteria, early identification and active intervention are essential to reduce the rate of critical illness and mortality and improve the cure rate. This is especially important for many patients whose symptoms are not typical. This study found that the incidence of an early decrease in the lymphocyte proportion and lymphocyte count in patients was higher in those showing severe illness subsequently, and CRP elevation at the early stage of hospitalization was positively correlated with the proportion of patients showing severe disease. In particular, the proportion of patients with early CRP levels greater than 40 was particularly high (over 50%). Therefore, early CRP values have great prognostic value.

Although the presence of Wuhan exposure history had no significant effect on the disease, both critically severe patients who died had Wuhan exposure history. Thus, the viral virulence in the first generation of cases with Wuhan exposure may have been stronger. The viral load in the body was not positively correlated with the severity of the disease, but when the patient developed pneumonia, the viral load in the body was significantly reduced [14]. Because the blood biochemical indicators are related to the severity of the disease, early detection of the severity of disease through assessment of blood biochemical indicators and selection of early intervention options according to the situation will be helpful for disease control and prognosis. When health systems are overburdened, CRP evaluations and routine blood tests to observe the lymphocyte percentage and count can serve as low-cost and rapid approaches for disease evaluation. Patients with mild disease can be temporarily treated in isolation at home.

Nanfang hospital of Southern Medical University of China jointly released the results of a study [15] that mainly addressed the factors affecting the mortality risk of patients in the early stage of the new crown pneumonia outbreak. The results showed that the patient's age and CRP levels were independent risk factors for predicting death, i.e., age and CRP predicted the risk of death in patients with COVID-19. This is consistent with the results of the multivariate regression analysis in this paper, which suggested that the early CRP levels can predict the severity of the disease. Although our study also showed that age affected the disease condition when other influencing factors were not considered, the multifactor regression analysis in this paper showed that age was not related to the disease condition. Unlike the results reported by Southern Medical University, comorbidities were shown to affect the severity of the condition. This may also be related to the small sample size of this study and needs to be confirmed by further research. The results presented above suggest that, for the population or confirmed patients whose nucleic acid test results are negative but whose performance is similar to that of the patients with COVID-19, patient age, the presence of complications, a significant decline in lymphocytes, a significant increase in the ESR, and a significant increase in CRP levels can be used to predict the risk of short-term death and strengthen management, rationally distribute medical materials, and reduce the conversion rate and death rate of patients with critical diseases.

A recent study published in *Lancet Respiratory Medicine* showed that [9] the combination of ground-glass shadows seen on imaging with corresponding pale lesions seen by the naked eye suggests that the new crown pneumonia mainly causes an inflammatory response characterized by deep airway and alveolar injury. This study also found that a reduced lymphocyte count is common in the early stages of disease (25%). In some cases, it is serious, and this finding is consistent with the results of two recent reports [16,17]. Since the time from symptom onset to development of acute respiratory distress syndrome (ARDS) is only 9 days in the initial COVID-19 patients [18], early identification of the disease is necessary for the treatment of these patients.

There are some obvious limitations to our research. First, this study only represents the characteristics of Changsha COVID-19 in the early stage, which is of certain value for prevention and treatment and research in the future. Thus, the time of data extraction was limited, the number of cases was limited, and the contact history and laboratory test records of some cases were incomplete. Second, because the patients discharged early were not included in the asymptomatic infected patients, the actual situation is that the patients who were followed up mostly had mild disease, which may affect the role of epidemiology in the prognosis of the disease. Third, due to the inconsistent enthusiasm of patients seeking medical treatment, the time nodes of all examination results cannot be unified, which may lead to some errors.

## 5. Conclusions

The presence or absence of comorbidities and CRP elevation were independent significant predictors of COVID-19 severity.

## Figures and Tables

**Table 1:** Baseline characteristics and laboratory findings of patients infected with 2019-nCoV on admission to hospital.

	All patients (*n* = 108)	Disease severity	
		Severe (*n* = 24)	Nonsevere (*n* = 84)
Age			
Median, year	43.61	54.46	40.44

Distribution			
0–＜14 yr	4 (3.7%)	0	4 (4.76%)
≥14–＜40 yr	42 (38.89%)	2 (8.33%)	40 (47.61%)
≥40–＜65 yr	48 (44.44%)	16 (66.67%)	32 (38.1%)
≥65 yr	14 (12.96%)	6 (25%)	8 (9.52%)

Sex			
Male	56 (51.85%)	12 (50%)	44 (52.38%)
Female	52 (48.15%)	12 (50%)	40 (47.62%)

Wuhan exposure			
Yes	58 (53.7%)	16 (66.67%)	42 (50%)
No	50 (46.3%)	8 (33.33%)	42 (50%)

Comorbidities			
Yes	35 (32.41%)	17 (70.83%)	18 (21.43%)
No	73 (67.59%)	7 (29.17%)	66 (78.57%)

Hypertension	16 (14.81%)	109 (41.67%)	6 (7.14%)
Diabetes	5 (4.63%)	2 (8.33%)	3 (3.57%)
Cerebral infarction	4 (3.7%)	3 (0.125%)	1 (1.19%)
Respiratory disease	3 (2.78%)	2 (8.33%)	1 (1.19%)
Liver disease	10 (9.26%)	4 (16.67%)	6 (7.14%)

Symptoms			
Fever	73 (67.59%)	23 (95.83%)	50 (59.52%)
Cough	52 (48.15%)	13 (54.17%)	39 (46.43%)
Pharyngalgia	15 (13.89%)	2 (8.33%)	13 (15.48%)
Expectoration	11 (10.19%)	3 (12.5%)	9 (10.71%)
Dyspnea	8 (7.41%)	7 (29.17%)	1 (1.19%)
Dizziness	5 (4.63%)	2 (8.33%)	3 (3.57%)
Headache	4 (3.7%)	3 (12.5%)	1 (1.19%)
Weakness	40 (37.04%)	13 (54.17%)	27 (32.14%)
Myalgia	12 (11.11%)	6 (0.25%)	6 (7.14%)
Chills	10 (9.26%)	2 (8.33%)	8 (9.52%)
Diarrhea	5 (4.63%)	3 (12.5%)	2 (2.38%)

Laboratory check (normal value) White blood cell count (4–10 × 10^9^/L)			
<4	39 (36.11%)	6 (25%)	33 (39.29%)
≥4–＜10	65 (60.19%)	17 (70.83%)	48 (57.14%)
≥10	4 (3.7%)	1 (4.17%)	3 (3.57%)

Lymphocyte ratio (20–40%)			
<20	27 (25%)	11 (45.83%)	16 (19.05%)
≥20–≤40	74 (68.52%)	13 (54.17%)	61 (72.62%)
>40	7 (6.48%)	0	7 (8.33%)

Lymphocyte count (0.8–4.0 × 10^9^/L)			
<0.8	25 (23.15%)	11 (45.83%)	14 (16.67%)
≥0.8–≤4.0	81 (75%)	13 (54.17%)	68 (80.95%)
>4.0	2 (1.85%)	0	2 (2.38%)

CRP (0–8.0 mg/L)			
≤8	32 (29.63%)	0	32 (38.1%)
>8–≤20	26 (24.07%)	7 (29.17%)	19 (22.62%)
>20–≤40	29 (26.85%)	6 (25%)	23 (27.38%)
>40	21 (19.44%)	11 (45.83%)	10 (11.90%)

ESR (0–15 mm/h)	*n* = 103	*n* = 21	*n* = 82
≤40	54 (52.43%)	5 (23.81%)	49 (59.76%)
>40–≤80	39 (37.86%)	13 (61.9%)	26 (31.71%)
>80	10 (9.71%)	3 (14.29%)	7 (8.54%)

## Data Availability

The data used to support the findings of this study are available from the corresponding author upon request.
